# Ultrasound Assisted Catheter-Directed Thrombolysis of Acute Pulmonary Embolism: A Review of Current Literature

**DOI:** 10.7759/cureus.1492

**Published:** 2017-07-19

**Authors:** Muhammad A Mangi, Hiba Rehman, Vikas Bansal, Omer Zuberi

**Affiliations:** 1 GME Internal Medicine, Orange Park Medical Center; 2 Critical Care Medicine, Mayo Clinic Jacksonville, Fl; 3 Cardiology, Orange Park Medical Center

**Keywords:** ultrasound assisted thrombolysis, ekos, pulmonary embolism

## Abstract

Pulmonary embolism continues as a very common and also presumably life-threatening disorder. For affected individuals with intermediate- as well as high-risk pulmonary embolism, catheter-based revascularization procedures have developed a possible substitute for systemic thrombolysis or for surgical embolectomy. Ultrasound-assisted catheter-directed thrombolysis is an innovative catheter-based approach; which is the main purpose of the present review article. Ultrasound-assisted catheter-directed thrombolysis is much more efficacious in reversing right ventricular dysfunction as well as dilatation in comparison to anticoagulation alone in individuals at intermediate risk. However, a direct comparison of ultrasound-assisted thrombolysis with systemic thrombolysis or surgical thrombectomy is not available. Ultrasound-assisted thrombolysis with early intrapulmonary thrombolytic bolus could also be successful in high-risk patients, but unfortunately, data from randomized trials is limited. This review article recapitulates existing information on ultrasound-assisted thrombolysis for acute pulmonary embolism.

## Introduction and background

Pulmonary embolism (PE) is one of the leading cause of mortality in the United States (US), accounting 5-10 % in-hospital mortality, 15% 90 days mortality and 58% mortality in the first hour in patients with hemodynamic compromise [1-3]. Pulmonary embolism results from occlusion of pulmonary trunk, main pulmonary artery, segmental or subsegmental branches by air, tumor, or blood clot. Symptoms and outcomes vary based on the severity and extent of occlusion. Pulmonary embolisms are categorized as small (low risk), sub-massive (intermediate risk) and massive (high risk). Small PEs does not cause right ventricular dysfunction and no hemodynamic compromise. Sub-massive PEs causes right ventricular dysfunction but no hemodynamic compromise. While massive PEs causes right ventricular dysfunction and hemodynamic compromise.

Massive and submassive PEs are associated with increased risk of morbidity and mortality and may require interventions to prevent the complication of PEs such as right heart failure and pulmonary artery hypertension (PAH) [4-7]. Systemic thrombolysis and surgical embolectomy carry a higher risk of complications. Systemic thrombolysis accounts for 20% risk of major hemorrhage and 3-5% risk of hemorrhagic stroke [2,8], while surgical embolectomy accounts 27.2% inpatient mortality rate [9]. Hence these interventions are limited to massive PE. However, for submassive PEs, ultrasound assisted catheter-directed thrombolysis (UACDT) has given us the promising results [10-11]. EkoSonic Endovascular System (EKOS Corporation, Bothell, Washington, United States) facilitates thrombolysis with the lower risk of bleeding and was tested in randomized clinical trials (RCTs) [12-13]. In one RCT, the UACDT was superior to heparin alone in reversing the right ventricular dysfunction at 24 hours without increased risk of bleeding in intermediate risk patients [12]. While in another trial UACDT improves the thrombus burden, right ventricular dilatation, and PAH with a lower risk of intracranial hemorrhage in both massive and submassive PEs [13]. It is because of these successful result, US Food and Drug Administration (FDA) approved the use of UACDT in patients with PE [14].

This review will elaborate current evidence and research on risk stratification of PE, UACDT safety, efficacy, limitation, and complications.

## Review

Risk stratification

PE risk stratification is important as severity and outcome of PE depends on the risk and comorbidity. The patient can be asymptomatic or symptomatic with dyspnea, syncope or chest pain. Similarly, the treatment also varies on the severity of PE. For low-risk PE anticoagulation and early discharge is recommended and for intermediate to high-risk PE intensive care unit (ICU) admission and intervention is recommended. Risk and severity are determined on the basis of certain characteristics including right heart strain, clot burden, elevated cardiac enzymes or brain natriuretic peptide (BNP) or N-terminal pro b-type natriuretic peptide (NT-pro-BNP) and hypotension [15-16].

*High Risk/ Massive Pulmonary Embolism: *Patients with massive PE usually present with syncope, dyspnea, hypoxemia, bradycardia, and hypotension. They have large clot burden, a residual clot in heart or clot in the proximal part of lower extremity veins like Iliofemoral veins. Due to clot burden, these patients often have hypotension and right heart strain, which makes higher cardiac enzymes, BNP, and proBNP. These types of patients are at high risk of decompensation and death and often required emergent interventions and ICU admission.

*Intermediate Risk/ Submassive Pulmonary Embolism: *Patients with sub-massive PE are hemodynamically stable, but they have right heart strain which makes higher cardiac enzymes, BNP and pro-BNP. They are at increased risk of decompensation and death [16]. They also required intervention and ICU admission to prevent decompensation and death.

*Low Risk/ Small Pulmonary embolism:* Patients with small PE are hemodynamically stable with no right ventricular strain. Patients with hypotension, right ventricular strain, history of coronary artery disease, residual deep vein thrombosis, PE in the central location, and hypoxia are the independent risk factors for clinical deterioration [17]. The absence of mentioned risk factor often labels the patient as a low risk which often requires anticoagulation and early hospital discharge [17].

Ultrasound-assisted catheter-directed thrombolysis

**Figure 1:**
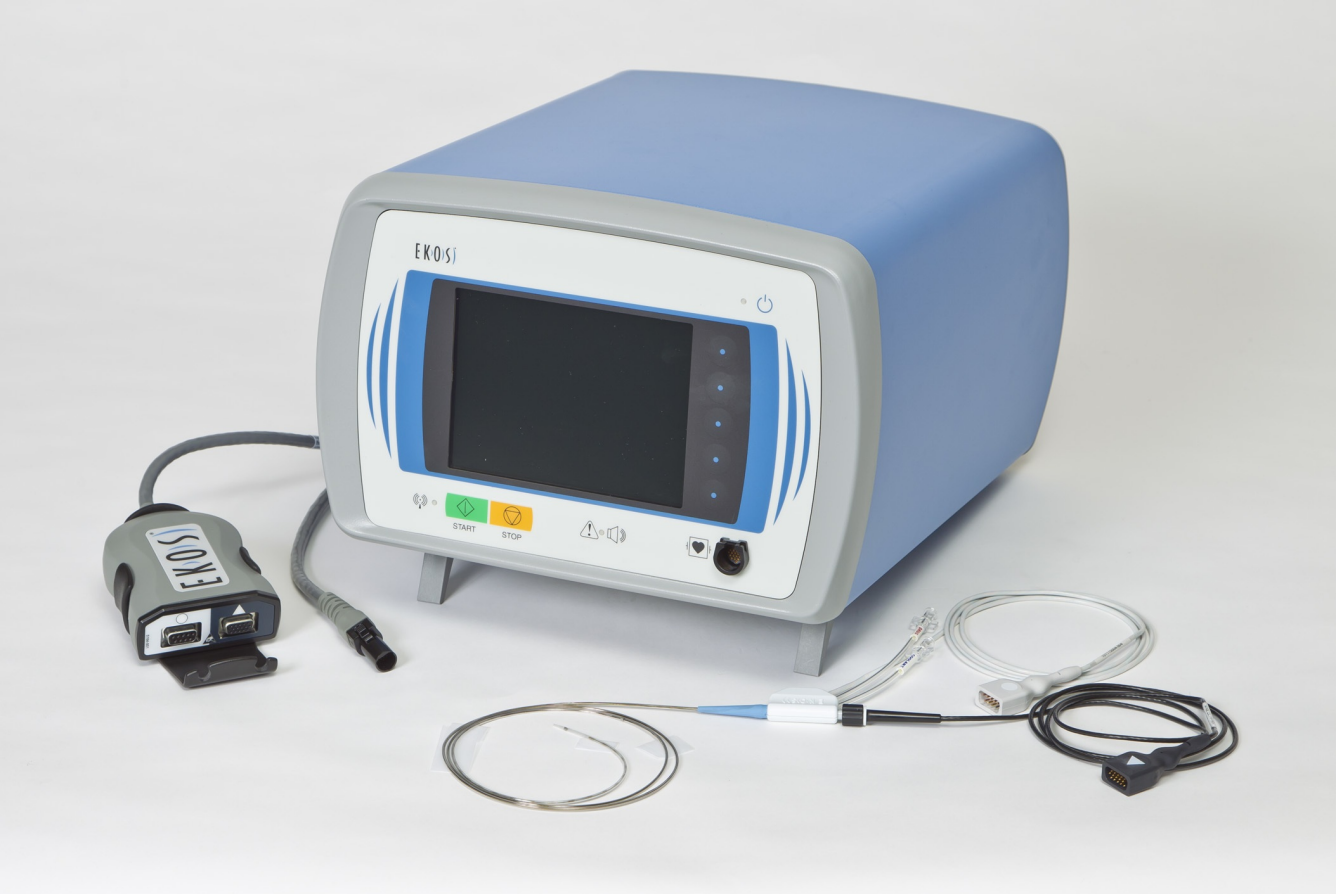
EkoSonic device with control unit and catheters. (Reproduced with permission of the EKOS Corporation, Bothell, Washington, United States)

**Figure 2:**
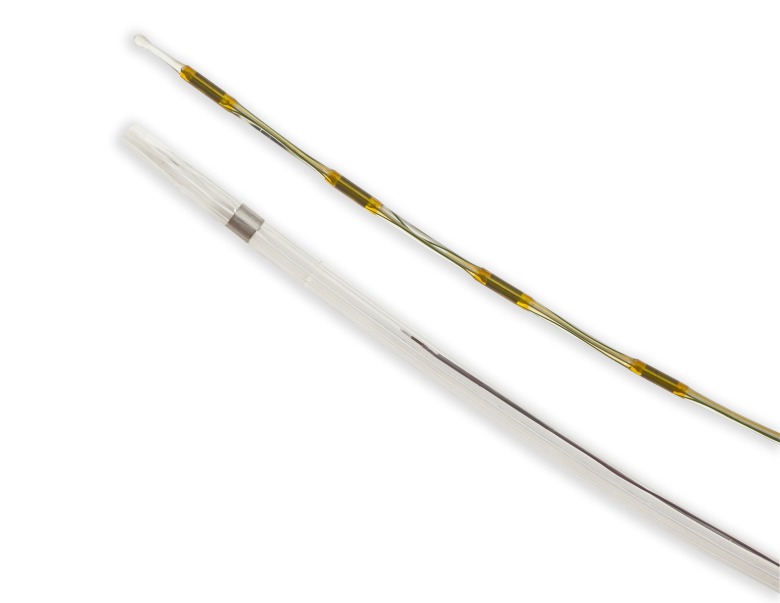
Closer view of the catheters (reproduced with permission of the EKOS Corporation, Bothell, Washington, United States)

UACDT is a pharmaco-mechanical thrombolysis consists of a catheter system which deploys ultrasound waves into the blood clots [18]. Ultrasound facilitates the delivery of the thrombolytic agent into the blood clots [19-21]. Further Braaten, et al. described the mechanism of ultrasound assisted thrombolysis (UAT) on blood clot [20]. Ultrasound causes reversible disaggregation of uncrosslinked fibrin fibers, which create additional binding sites, hence promotes thrombolysis [20]. Moreover, ultrasound waves create low power acoustic streaming, which also separates fibers to promote the penetration of the thrombolytic drug into the thrombus [19]. The EkoSonic Endovascular System (EKOS Corporation; Bothell, Washington, United States) is consists of three component, catheter which delivers the drug to the confined location, microsonic core wire which delivers high-frequency, low-energy intravascular ultrasound waves along the treatment area and control unit which control this whole process (Figure 1-2).

UACDT is an emerging field in the management of PE. Research is currently being conducted on the management of massive and submassive PE. In common, they used recombinant tissue plasminogen activator (rt-PA) as a thrombolytic agent. In some studies bolus of rt-PA was administered before UACDT [18,22]. Some studies used a fixed dose of rt-PA (10mg over 15 hours) per treated lung, while other used pulse infusion or slow infusion [12,18,23]. Treatment duration also varied among studies and outcome is guided by clinical or angiographic measures [11,24-26]. 

Efficacy and safety of US-guided thrombolysis

*Efficacy of Ultrasound Assisted Catheter-Directed Thrombolysis: *The most common parameter to guide treatment is right ventricular–to–left ventricular (RV/LV) ratio, which is a validated parameter to predict short-term mortality in patient with PE [6,27-28]. The studies have used this parameter to guide treatment of massive and submassive PE. In some studies, authors assessed RV/LV ratio before and after ultrasound-assisted thrombolysis (UAT) and they found a significant improvement of RV/LV ratio [12,18,22]. Engelhardt, et al. reported RV/LV ratio decreased from 1.33 ± 0.24 at baseline to 1.00 ± 0.13 at follow-up (p < 0.001) and modified Miller score was significantly decreased from 17.8 ± 5.3 at baseline to 8.7 ± 5.1 at follow-up (p < 0.001) [22]. The modified Miller score was used to assess pulmonary clot burden.

This gave us the promising result, as in randomized controlled Tenecteplase Italian Pulmonary Embolism (TIPES) trial, treatment group RV/LV ratio decrease from 1.36 to 1.03 [29]. In the treatment group, the patient received tenecteplase plus unfractionated heparin (UFH) were compared with the control group receiving UFH alone. The RV/LV ratio in the treatment group decreased from 1.36 to 1.04 over 24 hours compared to control group from 1.32 to 1.22. Other variables which improved in patients receiving UAT were pulmonary artery pressure (PAP), clot burden, tricuspid annular plane systolic excursion and cardiac index [12,18,30-33]. Later on Kennedy, et al. described the effectiveness of UACDT in the retrospective study [11]. There was ≥ 90% thrombus clearance in 57% of the patients and 50%-90% thrombus clearance in 41% of the patients with PE over 19.6 hours±6.0. The PAP was decreased from 47 mm Hg±15 at baseline to 38 mm Hg±12 (P <.001) and Miller score decreased from 25±3 at baseline to 17±6 (P <.001). However, long-term benefit of early revascularization is still under debate. Some studies suggested that early revascularization decreases the risk of chronic pulmonary artery hypertension [34-36]. The consistent evidence in support of UACDT efficacy leading to the beginning of the randomized controlled trial (RCTs).

The Ultrasound Assisted Catheter-Directed Thrombolysis for Acute Intermediate risk Pulmonary Embolism (ULTIMA) trial was the first RCT of UACDT [12]. In this trial, they compared whether UACDT with anticoagulation is superior to anticoagulation alone in patients with intermediate risk PE. The investigator's hypothesis was based on TIPES trial, they hypothesized that UACDT has a similar effect in improving RV/LV ratio compared to tenecteplase [28]. Patients with acute symptomatic PE located at least at main or lower lobe pulmonary artery with RV/LV ratio ≥ 1 were included. Total 59 patients were randomized (30 intervention group and 29 control group). The intervention group received rt-PA 10 mg per treated lung with UFH and control group received UFH only. In intervention group mean RV/LV ratio decreased from 1.28±0.19 at baseline to 0.99±0.17 at 24 hours (p <0.001) while in control group mean RV/LV ratio decreased from 1.20±0.14 to 1.17±0.20 (p=0.31). There was also a significant improvement in right ventricular systolic function in the intervention group. Subsequently Dumantepe, et al. evaluated the efficacy of UACDT in both massive and submassive PE and found ≥ 90% thrombus clearance in 77.2% of the patients, and 50% to 90% thrombus clearance in 22.8% of the patients [10]. They also discovered that PAP decreased from 67 ± 14 at baseline to 34 ± 11 mmHg, (p < 0.001) at follow-up, RV/LV ratio decreased from 1.29 ± 0.17 at baseline to 0.92 ± 0.11 at follow-up (p < 0.001) and modified Miller score was significantly decreased from 28 ± 4 at baseline to 13 ± 5, (p < 0.001) in 95% patients who survived to discharge. Further Engelberger, et al. retrospectively analyzed the safety and efficacy of UACDT [18]. He demonstrated mean pulmonary artery pressure (PAMP) decreased from 37 ± 9 mmHg at baseline to 25 ± 8 mmHg at 15 h (P < 0.001), cardiac index increased from 2.0 ± 0.7 at baseline to 2.7 ± 0.9 L/min/m (P < 0.001), and RV/LV ratio decreased from 1.42 ± 0.21 at baseline to 1.06 ± 0.23 at 24 h (P < 0.001). Most of the hemodynamic benefit was found in high-risk PE and in those with symptom duration < 14 days. Similarly Bagla, et al. conducted both retrospective and prospective study and found interesting finding relating to UACDT [37]. The main pulmonary artery pressure significantly decreased from 49.8 mm Hg at baseline to 31.1 mm Hg (P < .0001) and RV/LV ratios decreased from 1.59 at baseline to 0.93 (P < .0001). These findings further lead to the beginning of another clinical trial. In Single-arm, Multi-center Trial of EkoSonic® Endovascular System and Activase for Treatment of Acute Pulmonary Embolism (SEATTLE II) trial, there were 150 patients (Acute massive 31, sub-massive 119) included in this study [13]. 24 mg of rt-PA administered either as 1 mg/h for 24 h with a unilateral catheter or 1 mg/h/catheter for 12 h with bilateral catheters. Mean RV/LV diameter ratio decreased from baseline 1.55 to 1.13 at 48 h post-procedure (p < 0.0001) and PAMP decrease from 51.4 mm Hg to 36.9 mm Hg (p < 0.0001). They also observed decrease thrombus burden in patients treated with UACDT. It is because of these successful results, the use of UACDT was approved by the US FDA in May 2014 for the treatment of PE [14]. Further, in other retrospective studies, authors described other variables which significantly improved in UACDT, including pulmonary artery systolic, diastolic and mean pressure, main pulmonary artery diameter, tricuspid annular plane systolic excursion and Qanadli score [38,39]. Ozcinar, et al. in the prospective observational study also demonstrated the significance of UACDT in the patient with PE [40]. There were 38 patients enrolled in this study and 25 patients had bilateral UACDT with median rt-PA 21mg over median 15 hours. The median RV/LV ratio decreased from 0.9(0.7-1.1) at baseline to 0.7 (00.97), (p=0.001) at six months follow-up and PAP decreased from 61.4 ±16.7 to 37.2±9.1 mmHg (p=0.001). These findings collectively lead UACDT a better method of treating PE (Table 1). When compared to catheter-directed therapy alone, the UACDT provides similar efficacy but reduced time thrombolytic infusion time and treatment-related complications [19].

**Table 1:** Table representing the summary of studies published for efficacy of ultrasound assisted catheter directed thrombolysis UACDT= Ultrasound assisted catheter directed thrombolysis, CDT= Catheter directed thrombolysis, PAP= Pulmonary artery pressure, PAMP= Mean pulmonary artery pressure RVSF= Right ventricular systolic function, tPA= Tissue plasminogen activator, RV= Right ventricular, RA= right atrium, LA= Left atrium, UFH= Unfractionated Heparin, GUSTO = Global Utilization of Streptokinase and Tissue Plasminogen Activator for Occluded Coronary Arteries, CD= Catheter directed, CI = Cardiac index, TRV= Tricuspid regurgitation jet velocity, TAPSE= Tricuspid annular plane systolic excursion, ST= systolic myocardial velocity on tissue Doppler

Authors	Study Design	Number of patients	Massive/Submassive PE	Study details	Thrombolytic detail	RV/LV ratio before and after	Other outcome variables improved
Dumantepe, et al. 2014 [10]	Retrospective	Total 22, but 19 patients have symptoms <14 days	5/14	UACDT 22	Median dose of t-PA 21.0 mg (range 16 to 35 mg)	1.29 ± 0.17 vs 0.92 ± 0.11	Thrombus clearance (complete, near complete )PAP, modified Miller score
Kennedy, et al. 2013 [11]	Retrospective	60	12/48	UACDT 60	Total t-PA dose 35.1 mg	N/A	Thrombus clearance (complete, near complete, partial), PAP, Miller score
Kucher, et al. 2013 [12]	Prospective randomized clinical trial	59	0/59	UACDT 30, UFH 29	Total t-PA 10-20mg	1.28±0.19 vs 0.99±0.17	RV systolic function, TAPSE, PAP, PAMP, RA mean pressure, CI
Piazza, et al. 2014 [13]	Prospective randomized clinical trial	150	31/119	UACDT 150	Total t-PA dose 24mg	1.55 vs. 1.13	PAMP, modified Miller index score, GUSTO score
Engelberger, et al. 2013 [18]	Retrospective	52	14/32	UACDT 52	Total t-PA dose 22.0+9.1 mg for high-risk PE patients and 20.1+3.7 mg for intermediate-risk PE	1.42 ± 0.21 vs 1.06 ± 0.23	CI, PAMP
Engelhardt, et al. 2011 [22]	Retrospective	24	5/19	UACDT 24	Mean t-PA dose 33.5 ± 15.5mg	1.33 ± 0.24 vs 1.00 ± 0.13	modified Miller score
Al-Hakim, et al. 2014 [23]	Retrospective	18	0/18	UACDT 12, CDT 8	In UACDT total t-PA dose 20mg; In CDT total t-PA dose 23.7mg	N/A	PAP, RVSF, Clot clearance
Lin, et all. 2009 [24]	Retrospective	25	25/0	UACDT 11, CDT 14	In UACDT mean total t-PA dose of 17.2 ± 2.36 mg (range 8 –28 mg) In CDT mean total t-PA dose of 25.43 ± 5.27 mg (range 16–45 mg)	N/A	Thrombus clearance (complete, partial), Miler score
Chamsudiin, et al. 2008 [25]	Retrospective case series	10	10/0	UACDT 10	Mean dose of t-PA 0.88 mg/h +/- 0.19	N/A	Thrombus clearance (complete, near complete, partial)
Quintana, et al. 2013 [26]	Retrospective	10	2/8	UACDT 10	Total t-PA dose 18.0 mg	N/A	RVP, Mastora obstructive indices
Fuller, et al. 2017 [28]	Retrospective	27	0/27	UACDT 27	Total t-PA dose 12-24mg	Mean RV/LV reduction was 36.9% (±16.1%)	-
McCabe, et al. 2015 [30]	Retrospective	53	0/53	UACDT 53	Total t-PA dose 24.6 ± 9 mg	1.12±0.30 vs 0.98±0.20	Systolic PAP, PAMP
Ozmen, et al. 2015 [31]	Retrospective	10	0/10	UACDT 10	Total t-PA dose 31.7 mg ± 3.22	1.26 (0.76-1.84) vs 0.91 (0.62-1.10)	TRV, TAPSE, pulmonary acceleration time, PAP, Qandali score, RV dysfunction, clot burden
Nykamp, et al. 2015 [32]	Retrospective	45	12/33	UACDT 45	Total t-PA dose 30.5mg(range, 14-66mg)	N/A	Hypotension, tachycardia, cardiac dysfunction, PAP
Sag, et al. 2016 [33]	Prospective uncontrolled	13	13/0	UACDT 13	Total dose of t-PA 31.2 ± 15.3 mg	1.21±0.19 vs 1.01±0.14	RA diameter, RV diameter RA/LA ratio, TAPSE, PAP(systolic, diastolic, mean) Cardiac output, RA pressure
Bagla, et al. 2015 [37]	Prospective and retrospective uncontrolled study	45 (15 prospective, 30 retrospective)	0/45	UACDT 45	Total t-PA dose 24mg	1.59±0.54 vs 0.93±0.17	PAP
Kaymaz, et al. 2017 [38]	Retrospective	75	15/60	UACDT 75	Total t-PA dose 35.4 + 16.7	1.38±0.26 vs 1.17±0.19	PAP(mean, systolic, diastolic), TAPSE, RA/LA ratio, Qandali score, TAPSE, ST, PA diameter(main, right, left)
Liang, et al. 2016 [39]	Retrospective	63	8/55	UCADT 36 CDT 27	In UACDT total t-PA dose 27.5 + 12.9 mg; In CDT total t-PA dose 23.2 + 13.7 mg;	1.13±0.19 vs 0.77±0.19	TAPSE, PAP (systolic), RV dilation, RV systolic function
Ozcinar, et al. 2017 [40]	Prospective uncontrolled	45	N/A	UACDT	Total t-PA dose 21mg	0.9(0.71.1) Vs 0.7(00.97)	PAP, BNP
Kuo, et al. 2015 [41]	Prospective uncontrolled study	101	28/73	UACDT 36, CDT 64, CD mechanical thrombectomy 1	Total t-PA dose 28.0±11.0 mg	N/A	PAP, right heart strain

Safety of ultrasound assisted catheter-directed thrombolysis

The majority of literature and studies related to UACDT safety is assessed by five major variables including in-hospital mortality, 30 days mortality, 90 days mortality, major bleeding and minor bleeding. Engelhardt, et al. in retrospective study observed no mortality in patients with PE, and all patients were discharged alive [22]. There was not even a single major systemic bleeding complication. However, there were four access site major bleeding complications requiring transfusion and one suspected massive PE recurrence. Similarly, in another study, there were 57/60 patients survived to discharge (one patient died due to intra-abdominal hemorrhage and hypovolemic shock, one patient died due to family opted for comfort care measures and one died due to massive PE) [11]. There was one 90 days mortality during infusion of rt-PA, one patient had an acute renal injury and cardiopulmonary arrest, and one major and minor bleeding episode. This evidence made a consistent result with ULTIMA trial for the safety of UACDT procedure for PE. In ULTIMA trial, there was no death reported but there were three minor bleeding episodes (two with transient hemoptysis without intervention and one with access site hematoma managed with manual compression) in patients with intervention group and one minor bleeding episode (muscular hematoma at injection site of UFH) in patient with control group, and there was no reported episode of recurrent PE seen in either group. Further Engelberger, et al. reported two death during three-month follow-up (one from cardiogenic shock and one from recurrent PE) and two major bleeding episode (one intrathoracic bleeding after cardiopulmonary resuscitation requiring transfusion, one intrapulmonary bleeding requiring lobectomy), but there was no in-hospital mortality reported [18]. Moreover, in SEATTLE II trial total seven mortality (three in-hospital and rest 30-day mortality), 15 major bleeding events within 30 days (14 moderate and one severe bleeding), and five serious adverse events (device related and two rt-PA related) were described [13]. It is because of this consistent safety result the US FDA approved the use of UACDT in the patient with PE. Further in Pulmonary Embolism Response to Fragmentation, Embolectomy, and Catheter Thrombolysis (PERFECT) registry UACDT appears to be safe and effective without any major systemic bleeding including intracranial hemorrhage [41]. There was no intracranial hemorrhage reported with the use of UACDT until recently published case report [42]. But this patient was elderly male of 86 years along with thrombocytopenia on the day patient developed intracranial hemorrhage. It is uncertain whether this bleeding was solely due to use UACDT or multifactorial, considering old age and thrombocytopenia along with rt-PA during the procedure [42].

There was one intracranial hemorrhage reported by Ozcinar, et al. in the prospective observational study [40]. The author could not find any other study of UACDT who had an Intracranial hemorrhage. Overall there is lesser major bleeding event and mortality event with more benefit which suggests better safety of UACDT (Table 2). 

**Table 2:** Table representing the summary of studies published for safety of ultrasound assisted catheter directed thrombolysis N/A = Not available, PE= Pulmonary embolism *4= 3 in-hospital and 1 after discharge within 90 days.

Authors	In-hospital mortality	30 days mortality	90 days mortality	Major systemic bleeding episodes in UACDT	Other bleeding episodes in UACDT	PE recurrence in UACDT during follow up
Dumantepe, et al. 2014 [10]	1	0	0	0	2	1
Kennedy, et al. 2013 [11]	3	0	4*	1	1	0
Kucher, et al. 2013 [12]	0	0	1	0	3	0
Piazza, et al. 2014 [13]	3	4	N/A	12	3	N/A
Engelberger, et al. 2013 [18]	0	0	2	2	11	1
Engelhardt, et al. 2011 [22]	0	0	0	0	6	1
Al-Hakim, et al. 2014 [23]	0	N/A	N/A	0	1	N/A
Lin, et all. 2009 [24]	1	N/A	N/A	0	0	N/A
Chamsudiin, et al. 2008 [25]	0	0	0	0	2	N/A
Quintana, et al. 2013 [26]	0	0	0	0	2	1
Fuller, et al. 2017 [28]	0	N/A	N/A	0	4	N/A
McCabe, et al. 2015 [30]	0	N/A	N/A	2	3	N/A
Ozmen, et al. 2015 [31]	1	0	0	0	0	0
Nykamp, et al 2015 [32]	0	0	0	0	0	0
Sag, et al. 2016 [33]	1	0	0	0	0	0
Bagla, et al. 2015 [37]	0	0	N/A	2	4	0
Kaymaz, et al. 2017 [38]	4	N/A	N/A	2	5	0
Liang, et al. 2016 [39]	1	N/A	N/A	2	3	N/A
Ozcinar, et al. 2017 [40]	0	N/A	N/A	2	2	0
Kuo, et al. 2015 [41]	6	0	0	0	13	N/A

Complications

There are numbers of complications related to UACDT, Including vascular access related complications (bleeding, hematoma, rupture of vessels), cardiogenic shock, pulmonary hemorrhage, perforation or dissection of the pulmonary artery, and pericardial tamponade [43]. Other more common complications are arrhythmias and right sided valvular regurgitation [43]. Moreover, the use of contrast also leads to contrast-induced nephropathy [43]. Finally, the cost of the procedure compared to systemic thrombolysis as UACDT is more expensive than systemic thrombolysis [43]. Moreover Liang, et al. stated that there are no statistical differences in clinical, hemodynamic outcomes and procedural complication rates between UACDT and standard catheter-directed thrombolysis without ultrasound [39]. However this study had several important limitations including relatively smaller sample size, changing procedural protocol, and absence of post-procedure testing to demonstrate any improvement.

Limitations

It is unclear whether UACDT acts fast enough to prevent hemodynamic compromise and death in unstable patients. Other limitations are long duration procedure (15-24 hours), expensive procedure, limited availability in majority hospitals, unsure about the recurrence of PE, and chances of chronic thromboembolic PAH.

## Conclusions

Ultrasound-assisted catheter-directed thrombolysis (UACDT) is emerging and alternative revascularization procedure to systemic thrombolysis and surgical embolectomy for PE. UACDT is superior in improving RV/LV ratio, PAP, thrombotic burden and cardiac index with a lower risk of bleeding and other complications. UACDT procedure is performed in the limited hospital with around the clock availability of catheter therapy and surgical embolectomy. There is no head to head prospective randomized trial to compare UACDT with catheter-directed thrombolysis without ultrasound and UACDT with systemic thrombolysis. Further prospective randomized studies are warranted to compare the efficacy and safety of UACDT with catheter-directed thrombolysis without ultrasound and systemic thrombolysis.
